# What do VR experiments teach us about time?

**DOI:** 10.3389/fpsyg.2022.1082844

**Published:** 2023-07-26

**Authors:** Andrew J. Latham, Alex Holcombe

**Affiliations:** ^1^Department of Philosophy and History of Ideas, Aarhus University, Aarhus, Denmark; ^2^School of Psychology, University of Sydney, Sydney, NSW, Australia

**Keywords:** virtual reality, temporal experience, IGUS, temporal passage, time

Gruber and Smith (2019) have conducted some interesting virtual reality (VR) experiments, but we think that these experiments fail to illuminate why people think that the present is special. Their experiments attempted to test a suggestion by Hartle (2005) that with VR one might construct scenarios in which people experience the *same present twice*. If that's possible, then it could give us a reason to think that when we experience the present as being special, that's not because it's *objectively* so. Instead, our experience of the present being special is a feature of having a psychology like ours. While we are sympathetic to the thought that there is no objective present, we do not think that these experiments give us a reason to think this. That said, VR experiments, such as Gruber and Smith's, hold much promise for being able to illuminate various aspects of our temporal psychology.

According to Hartle's (2005) IGUS model (which is meant to resemble entities like us) sensory information is routed to two kinds of processes: conscious processes *C*, which cause behavior, and unconscious processes*, U*, which construct a schematic representation of the environment. Hartle proposed that we experience the present as being special because of the sensory information at each time entering into *C*. For Gruber et al. (2020), the succession of sensory information entering into *C* underpins our experience of time passing. Our experience of time passing is *illusory* because it fails to be veridical with how things are physically in the world (according to leading theories in physics, there is no change in physical time. See Buonomano and Rovelli, forthcoming for an accessible discussion). We have a genuine experience of time passing but time itself does not physically pass.

Alternatively, it could be that we do not experience time passing at all, rather people who claim to have an experience of time passing have false beliefs about their experience (Miller et al., 2020). We don't think that the illusion vs. false-belief debate is critical here; on either account, it would be interesting if people's claims about their experiences change while participating in Gruber and Smith's (2019) VR experiments.

Based on his IGUS model, Hartle (2005) suggested that if people are induced to sensorily process information concurrently from both the present sensory feed and a feed from the recent past, then their experience of the present would change. More specifically, Gruber et al. thought that in this scenario, people might experience the *same present twice*. With sensory information from the past routed back into *C*, the thought is that we might be able to experience that information as being the present again. In their experiments (Gruber and Smith, 2019), participants wore VR goggles and shifted their gaze around a scene of someone arranging three rows of dominoes. Participants were encouraged to press a button and were informed that pressing it would change what they were seeing from the present feed to a past feed. When we say *present feed*, we just mean that they receive sensory information *via* the VR system regarding how things are currently around them, whereas *past feed* means that they receive delayed sensory information *via* the VR system. For example, the participant can switch from watching the present feed of the experimenter laying out a third row of dominoes to the past feed and see the second row of dominoes being laid out again.

After the virtual reality session, participants were asked two probe questions. The first was: “seeing the second row of dominoes again was just as real as the first time.” The second probe was: “during the VR replay of the second row of dominoes it seemed like I was ‘there.”' All participants (though how many participants were tested was not reported by Gruber et al.; we implore all future researchers to report the details of their testing protocols, participants, and results) responded affirmatively to both probe questions and Gruber et al. took this to mean that participants experienced the past snapshot as being present again. That is, Gruber et al. think that participants experienced both sensory feeds, the present feed and the past feed, as having equal status as being the present. If that's right, it gives us a reason to think that our experience of the present being special is *not* because of anything objective about time. After all, if participants' experience of the present being special was tracking an objective feature of time, then people should only experience at most one of these sensory feeds as being the present.

However, there is an alternative and more straightforward explanation of participant's responses to these probe questions. Participants in this experiment chose which feed is fed into *C*, and recognize the past feed shows events that they have already experienced before. We think that they conceptualize the past feed as a recording (which it in fact is), so what they experience as the present is them viewing a recording. This is a bit like when a person recalls a past event—rather than thinking that they are experiencing the past event as being present again, what they experience as the present is the recalling of a memory of a past event. We think the participants' responses to the probe questions are consistent with this explanation.

Participants agreed with the statement that events in the past feed appeared as real as the first time, and that it felt like they were there. This is meant to be evidence that people don't just experience events as being present when they are viewed in the present feed but also when they are viewed in the past feed. But when participants responded that things in the past feed seemed just as real as when they were in the present feed, this doesn't require that they experienced the event of seeing the second row of dominoes as being present twice. We can imagine someone who recalls a vivid memory of an event responding that the event in their mind seems just as real as when it occurred. All this tells us is that the VR environment is rich and immersive, not that people are experiencing events in the present and past feed as having equal status as an objective present.

Gruber et al. say that they didn't ask people if they felt they were “in the past” because no participant has ever been in the past; but this seems to be what we want to know! Knowing whether participants experience the same event as being the present twice requires knowing whether participants experience past events being present again. Of course, we are sympathetic to the thought that probing this directly would be problematic. Participant responses will no doubt be influenced by their knowledge that things are not that way, making people reluctant to report that they seem that way too.

An additional problem is raised by some of our own work on judgments about time—people have difficulty grasping different models of time and their implications. Sometimes over half a sample has to be dropped due to comprehension failures (e.g., Everett et al., forthcoming). As a general principle, caution is warranted when interpreting participant reports.

Perhaps future studies could coach people. Once it is explained that we can have genuine experiences of things being a certain way that are not veridical with how things are (such as experiencing a past event again as present), then affirmative responses might be more interpretable. The unsolicited responses by participants reported by Gruber and Smith (2019) suggest this could be a fruitful endeavor, but at present they too might be explained away due to comprehension failures. Take, for example, the moving-spotlight model of time (e.g., Cameron, 2015). According to this model, while past, present, and future times all exist, one time is “illuminated” as the objective present and which time is illuminated changes. To genuinely experience a time as being the present twice would require that a time be illuminated as the objective present twice. But likely no one thinks anything like this is occurring in these experiments (even if it's consistent with what participants report). The only reason that participants recognize they are experiencing something like the same event twice is because when they experience the event the first time, it was incorporated into their schematic representation of how things are/were! But, this also means that these reports are consistent with A-theoretic models of time, according to which there is an objective present.

To bring out this line of thought further consider a different set of critical probe questions. We wonder what people believe their action affordances are while viewing the present feed and past feed. Imagine that you are in the VR experiment and while in the present feed you watch the experimenter as they lay out a third row of dominoes. Now imagine switching to the past feed, does it seem open to you that you can stop the experimenter laying out the third row of dominoes? The motivating thought here is that if people are experiencing the past feed as the present, then affordances for action that were apparent when the event was in the present feed should be similarly apparent again (although this depends somewhat on what one thinks counts as experiencing as the present). But as we have already noted, that seems unlikely. When you experience the experimenter laying out the third row of dominoes in the present feed, you update your schematic representation of the world such that there is now a third row of dominoes. Switching to the past feed does not undo this.

This thought is entirely consistent with Hartle's IGUS model. Our experience of the present is not just a function of the current sensory information being taken from the world but also our schematic representation of the world constructed from past sensory information. When the past feed is being fed into *C* it has already been incorporated into the schematic representation of the world. Thus, our present experience (or at least how it seems to us), while being similar to when the event was experienced for the first time, is nevertheless quite different from it. The consequences of the event in memory are already part of your schematic representation of the world and so the affordances that were present when the event was first experienced are also not the same. For example, if the experimenter knocks over the third row of dominoes in the present feed and then I switch to the past feed, I don't then think that I have an affordance to knock over the dominoes. My schematic representation of the world already represents things such that the affordance to knock over the dominoes is not available.

In summary, we suspect that when participants switch to the past feed, they experience it as the recording that it is, rather than as an objective present. We do not think that VR experiments are well placed to answer questions about the nature of time itself (see also Buonomano and Rovelli, forthcoming). However, we do think that they provide a powerful means of probing our temporal psychology. Immersive VR experiments could be used to adjudicate between different accounts of the mechanisms and processes responsible for our temporal beliefs and experiences, including what could cause people to make erroneous statements about their own temporal phenomenology.

## Author contributions

All authors listed have made a substantial, direct, and intellectual contribution to the work and approved it for publication.
